# Oxidative stress-dependent changes in immune responses and cell death in the substantia nigra after ozone exposure in rat

**DOI:** 10.3389/fnagi.2015.00065

**Published:** 2015-05-05

**Authors:** Selva Rivas-Arancibia, Luis Fernando Hernández Zimbrón, Erika Rodríguez-Martínez, Perla D. Maldonado, Gabino Borgonio Pérez, María Sepúlveda-Parada

**Affiliations:** ^1^Laboratorio de estrés Oxidativo y Plasticidad Cerebral, Departamento de Fisiología, Facultad de Medicina, Universidad Nacional Autónoma de MéxicoMéxico, México; ^2^Laboratorio de Patología Vascular Cerebral, Instituto Nacional de Neurología y Neurocirugía, Manuel Velasco SuárezMéxico, México

**Keywords:** inflammation, oxidative stress, ozone, substantia nigra, neurodegeneration

## Abstract

Parkinson's disease has been associated with the selective loss of neurons in the substantia nigra pars compacta. Increasing evidence suggests that oxidative stress plays a major role. The resulting increase in reactive oxygen species triggers a sequence of events that leads to cell damage, activation of microglia cells and neuroinflammatory responses. Our objective was to study whether chronic exposure to low doses of ozone, which produces oxidative stress itself, induces progressive cell death in conjunction with glial alterations in the substantia nigra. Animals were exposed to an ozone-free air stream (control) or to low doses of ozone for 7, 15, 30, 60, or 90 days. Each group underwent (1) spectrophotometric analysis for protein oxidation; (2) western blot testing for microglia reactivity and nuclear factor kappa B expression levels; and (3) immunohistochemistry for cytochrome c, GFAP, Iba-1, NFkB, and COX-2. Our results indicate that ozone induces an increase in protein oxidation levels, changes in activated astrocytes and microglia, and cell death. NFkB and cytochrome c showed an increase until 30 days of exposure, while cyclooxygenase 2 in the substantia nigra increased from 7 days up to 90 days of repetitive ozone exposure. These results suggest that oxidative stress caused by ozone exposure induces changes in inflammatory responses and progressive cell death in the substantia nigra in rats, which could also be occurring in Parkinson's disease.

## Introduction

In densely populated areas, higher ozone (O_3_) concentrations exist, inducing measurable transient changes in lung function and airway inflammation, aggravating pre-existing respiratory diseases such as asthma and resulting in excess mortality. In adults and children, the potential effects of ozone exposure include encephalopathic signs and central nervous system symptoms, cognitive effects, increased blood pressure and reduced measures of intelligence (Genc et al., 2012). Several reports have suggested that these types of symptoms could be produced by an altered redox state. However, the effect of exposure to low doses of ozone in specific areas of the brain has not been thoroughly studied.

Redox balance is necessary for the maintenance of homeostasis in organisms because redox signals participate in the regulation of many cellular pathways (Adibhatla and Hatcher, 2010). The loss of the oxidation-reduction balance causes an oxidative stress state; this state is characterized by an increase in pro-oxidants and a decrease in the capacity of the antioxidant system to resist the effects of the reactive oxygen species (ROS) and reactive nitrogen species (RNS) (Rivas-Arancibia et al., 2010; Halliwell, 2012). This is very important because in the chronic oxidative stress state, alterations of redox signaling incite the loss of regulatory pathways. Some examples of these alterations have been reported, including obstruction of the antioxidant system, alterations in the immune system, dysregulation of inflammatory responses, changes in the cellular cycle, and the loss of neuronal repair mechanisms (Rivas-Arancibia et al., 2010).

Moreover, loss of the mechanisms that control oxide reduction produces alterations in the physiological responses involved in the repair of tissue damage in addition to all of the previous changes, such as the oxidation of biomolecules, nucleic acids, proteins, lipids, and carbohydrates. These changes also contribute to mitochondrial alterations and cell death (Rodríguez-Martínez et al., 2013). Consequently, oxidative stress has been implicated in the pathophysiology of many neurodegenerative diseases and aging (Fukagawa et al., 2000; Luo and Roth, 2000; Dorado-Martínez et al., 2001; Hsieh and Yang, 2013; Rodríguez-Martínez et al., 2013).

Ozone is the result of photochemical air pollution, and higher concentrations are reported in more densely populated cities. When ozone is inhaled, it produces ROS, and this overproduction of ROS could affect the nervous systems in two distinct ways. The first is by affecting the olfactory bulb and brain tissue directly (Colín-Barenque et al., 1999); and the second is through the respiratory system, where it overwhelms the antioxidant defense system of the lungs in a dose-dependent manner. Finally, the ROS and RNS that are secondarily produced by ozone exposure can then reach the central nervous system (CNS) through the bloodstream, producing oxidative stress, (Dorado-Martínez et al., 2001) cellular damage (Angoa-Pérez et al., 2006; Pereyra-Muñoz et al., 2006) and increased lipid peroxidation in various brain structures (Rivas-Arancibia et al., 2000, 2003). Ozone has also been demonstrated to produce functional changes and cumulative structural damage in rats and monkeys exposed to O_3_ at levels similar to those found in currently occurring ambient peaks. Indeed, some previous reports have indicated that humans are likely to be more sensitive to O_3_ than rats (Lippmann, 1989). In addition, previous studies have reported that chronic exposure to ozone causes neurodegeneration in the striatum and substantia nigra after 30 days of exposure to 0.25 ppm ozone (Pereyra-Muñoz et al., 2006). The substantia nigra and striatum are particularly vulnerable to oxidative stress because normal dopamine metabolism involves many oxidative reactions (Hermida-Ameijeiras et al., 2004). Moreover, ozone exposure increases the production of dopamine quinones, oxidative metabolites of dopamine, and the inactivation of antioxidant systems (Rosengren et al., 1985). Ozone exposure also alters redox signals, which contributes to the increase of oxidative stress (Santiago-López et al., 2010) and the activation of pathways that cause dopaminergic neuronal death (Pereyra-Muñoz et al., 2006). Damage and cellular death contribute to an increase in oxidative stress and the inflammatory response, which cause alterations in the blood-brain barrier (Mosley et al., 2006). Dopamine oxidation has a direct correlation with dopaminergic neuronal death in the substantia nigra (Santiago-López et al., 2010).

We developed a noninvasive animal model of the oxidative stress state by exposing rats to daily low doses of ozone (0.25 ppm). We have previously reported that ozone causes progressive neurodegeneration depending on the dose of chronic exposure to this gas (4).

Our aim was to determine if chronic and repetitive exposure to low doses of ozone causes oxidative stress, progressive neurodegeneration and dysregulation of inflammatory responses.

## Materials and methods

### Animals and animal care

Seventy-two (72) male Wistar rats, weighing 250–300 g, were individually housed in acrylic boxes with food provided *ad libitum* (NutriCubo, Purina, USA). Acrylic boxes were kept in a clean-air box. Both the control and treated rats were maintained in a temperature- and humidity-controlled environmental bioterium. The animals were kept and treated in accordance with the Norma Official Mexicana NOM-036-SSA 2- 2002, the National Institutes of Health Guidelines for Animal Treatment and the ethical committee of the faculty of Medicine at the National Autonomous University of Mexico.

### General procedures

Rats were randomly divided into six experimental groups (*n* = 12 per group). Group 1 was composed of animals exposed daily to a clean air stream free of O_3_for 4 h, and groups 2, 3, 4, 5, and 6 were animals exposed for 7, 15, 30, 60, and 90 days, respectively, to O_3_. The experimental groups were exposed daily to 0.25 ppm ozone for 4 h. (Halliwell, 2012)

### O_3_ exposure

Each day, animals were placed inside a chamber with a diffuser connected to a variable flux ozone generator (5 L/s) for 4 h. The procedure used has been previously described (Pereyra-Muñoz et al., 2006; Rivas-Arancibia et al., 2010). Previously filtered air was used by the ozone generator to produce ozone. Ozone production levels were proportional to current intensity and air flux. A PCI Ozone and Control System Monitor (W. Caldwel-N.J., USA) was used to measure the ozone concentration inside the chamber throughout the experiment and to keep the ozone concentration constant.

A similar chamber was used for the control group, for which ozone-free air was administered for 30 days.

Two hours after the last exposure to clean air or O_3_, animals from each group were deeply anesthetized with sodium pentobarbital (50 mg/kg i.p.; Sedalpharma, Edo. de México, México) and killed by decapitation. Samples of plasma were obtained for spectrophotometry assays. Finally, the substantia nigra of six animals from each group were obtained for western blot, and the other six animals from each group were transcardially perfused with 4% paraformaldehyde (Sigma-Aldrich Chemie, Germany) in 0.1 M phosphate buffer (J.T Baker, NJ) (PB, Tecsiquim; pH 7.4).

The brains were post-fixed with 10% formaldehyde (J.T Baker, USA) for 24 h and embedded in paraffin (McCormick, St. Louis, MO, USA). Sagittal brain slices (5 μm) containing the substantia nigra were obtained using a microtome (American Optical, model #680, clearence angle = 19°) and mounted on slides. For each group, sagittal brain slices containing the substantia nigra were processed for immunohistochemistry assays.

### Protein carbonyl content

Carbonyl formation was assessed based on the formation of the protein hydrazone by reaction with 2, 4-dinitrophenylhydrazine (DNPH). Plasma from the rats was incubated overnight with 10% streptomycin sulfate to remove nucleic acids and centrifuged at 21,000 g at 4°C for 40 min. The plasma samples were treated with 10 mM DNPH (in 2.5 M HCl) for 1 h at room temperature; 10% trichloracetic acid was added, and the samples were centrifuged at 2500 g at 4°C for 10 min. The pellets were washed three times with ethanol:ethyl acetate (1:1), dissolved with 6 M guanidine hydrochloride (in 20 mM phosphate buffer, pH 7.4), incubated for 10 min at 37°C, and centrifuged at 5000 g at 4°C for 3 min to remove insoluble material. Absorbance was measured at 370 nm. The protein carbonyl content was expressed as nmol carbonyl/mg protein using the molar absorption coefficient of DNPH (22,000 M-1 cm-1). The total protein concentration was obtained by comparison with the optical density at 280 nm of blank tubes prepared in parallel (only treated with HCl) and a standard curve of bovine serum albumin (0.25–2 mg/mL) prepared in 6 M guanidine-HCl.

### Western blot

The expression levels of Iba-1 and NFκB were analyzed by gel electrophoresis and western blot. The tissue was homogenized, and 50 μg of protein from each sample was boiled and separated on a 10% SDS polyacrylamide gel for 45 min. For WB analysis, proteins were electrophoretically transferred onto a PVDF membrane (Sigma-Aldrich). The membranes were blocked with 5% fat-free milk in Tris buffer solution (TBS-T) with 0.01% Tween 20 (TBS-T) (Sigma-Aldrich) for 2 h at 37°C to eliminate non-specific binding. After the blocking step, membranes were incubated individually with the Iba-1 and NFκB antibodies overnight under gentle shaking at 4°C (Brinkmann OrbMix 110, Brinkmann, Germany). Membranes were rinsed three times with TBS-T, incubated for 2 h at room temperature (RT) in TBS-T containing the HRP-conjugated anti-rabbit IgG secondary antibody diluted 1:10,000 with goat anti-rabbit IgG conjugated to horseradish peroxidase (1:10,000) (sc-2004 Biotechnology, Santa Cruz, CA) for 1 h and then rinsed three times with TBS-T. Immunoreactive bands were detected by chemiluminescence (ECL; General Electric, Santa Clara, CA). For densitometric analysis of Western blot images ImageJ software was used.

### Immunohistochemistry for cyt c, GFAP, Iba-1, NFKB and COX-2

Monoclonal mouse anti-glial fibrillary acidic protein (GFAP) for astrocytic cells and polyclonal mouse anti-ionized calcium-binding adapter molecule 1 (Iba-1) antibodies were obtained from Biocare. Rabbit polyclonal anti-cytochrome c, mouse polyclonal anti-nuclear factor kappa-B (NFκB) and rabbit polyclonal anti-cyclooxygenase 2 (COX-2) were obtained from Santa Cruz Biotechnology, CA, USA.

For each brain, sagittal sections containing the substantia nigra were treated with a paraffin-removal and heat-retrieval solution (Biocare Medical) and put into an electric pressure cooker (Decloaking Chamber, Biocare Medical) for 5 min. After that, slides were washed with distilled water and treated with 3% hydrogen peroxide (diluted 1:5; Fisher Scientific) for 5 min. Then, sections were rinsed with distilled water, treated with a blocking reagent (Background Sniper, 4plus Detection Component, Biocare Medical) for 10 min, washed with 0.1 M phosphate saline buffer (PBS; pH 7.4; Merck), and incubated for 12 h at 4°C with anti-cyt c (diluted 1:200), anti-GFAP (diluted 1:200), anti-Iba-1(diluted 1:300), anti-NFkB (diluted 1:200, Santa Cruz Biotechnology) and anti-COX-2 (diluted 1:200). Sections were rinsed with PBS and treated with biotinylated secondary antibody (Universal Link, Biocare Medical) for 1 h. After being washed with PBS, treated with streptavidin-enzyme conjugates (4 plus the detection component streptavidin-HRP, Biocare Medical) for 30 min, and washed again with PBS, the bounded antibody was visualized using 3, 3-diaminobenzidine (DAB Substrate Kit, ScyTek) as the chromogen. The slices were washed in distilled water and counterstained with hematoxylin-buffered solution. Representative brain sections from each group were processed in parallel after coverslipping with Permount. The sections were then examined with a BX41 Olympus Microscope and photographed with an Evolution-QImagin Digital Camera Kit (MediaCybernetics).

### Number of cells in the substantia nigra

Six animals from each experimental and control group were analyzed. The number of immunoreactive cells in the substantia nigra was counted using six representative brain sections from each group for each antibody. For each group, the total number of immunoreactive cells per microscopic field at 40X magnification was counted. The microscopic field had an area of 30,000 μm^2^ and was 5-μm thick). The number of cells per field was counted, and the median number of cells was calculated.

### Statistics

Protein peroxidation levels and cell number were expressed as medians and analyzed with Kruskal–Wallis and Mann–Whitney *U*-tests.

## Results

**Figure 1:**
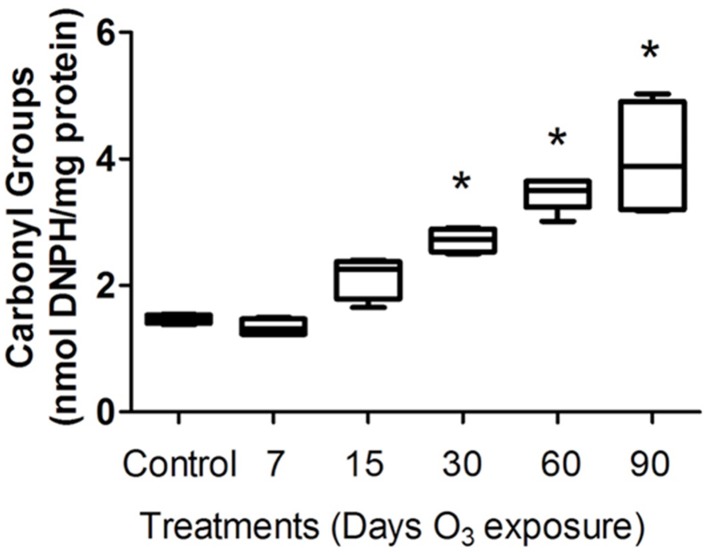
**Protein carbonyl levels in the plasma of rats exposed to ozone**. The ordinate represents carbonyl group levels, expressed as nmol DNPH/mg of plasma protein (mean). The treatments are indicated under each bar. ^*^Significantly different from the control group (vs. 30, 60, and 90 days of ozone exposure; *P* < 0.05).

To evaluate the oxidative injury caused by ozone exposure, we measured protein carbonyl content in the plasma of rats. The protein carbonyl formation in the plasma of rats increased progressively with the time of exposure to ozone in the 30- and 90-day treatment groups. The Kruskal–Wallis test showed significant between-group differences in the amount of protein oxidized (*P* < 0.0005). The Mann-Whitney *U*-test showed significant differences between the control group and the 30-day (2.43 ± 0.18 nmol DNPH/mg protein) (*P* < 0.05), 60-day (3.47 ± 2 nmol/mL) (*P* < 0.05) and 90-day (3.23 ± 1.007 nmol DNPH/mg protein) (*P* < 0.05) ozone-exposed groups. Protein carbonyl levels in the plasma progressively increased as a function of the duration of exposure (Figure 1).

## Immunohistochemistry

### Cytochrome c immunohistochemistry

**Figure 2:**
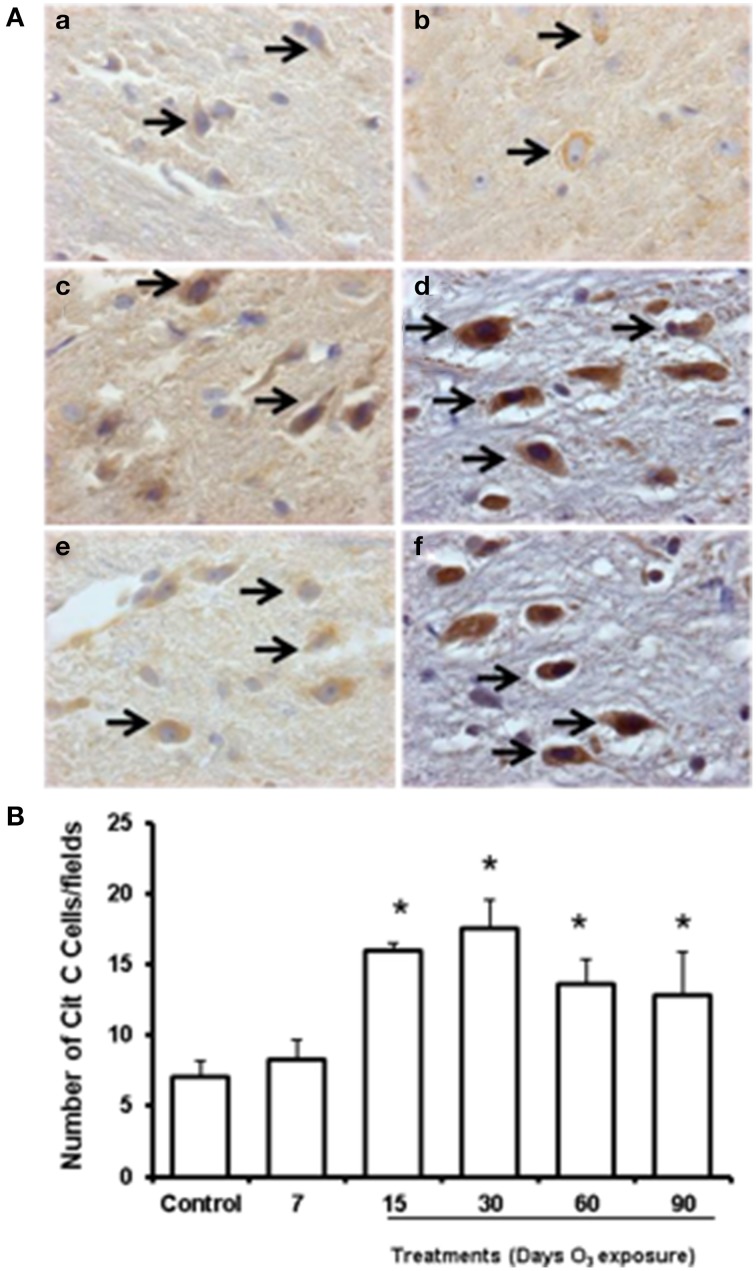
**Effect of ozone treatment on cytochrome c immunoreactivity in the substantia nigra**. **(A)** Light photomicrographs show cytochrome c immunoreactivity in the substantia nigra of the rats treated with air only (a), 7 days of ozone exposure (b), 15 days of ozone exposure (c), 30 days of ozone exposure (d), 60 days of ozone exposure (e), and 90 days of ozone exposure (f). Arrows show cytochrome c immunoreactivity in normal cells. 40X. We observed an increase in cytochrome c immunoreactivity and nucleus translocation (arrows in c, d, and f). The graph shows the effect of ozone treatment on the number of cytochrome c-positive cells in the substantia nigra **(B)**. The mean numbers of cytochrome c-positive cells are depicted on the ordinate. The treatments are indicated under each bar (control group and 7, 15, 30, 60, and 90 days of ozone exposure); *n* = 6 per group. ^*^P < 0.05.

To determine whether cytochrome c was released from mitochondria within the cytoplasm of substantia nigra cells and to test whether such release was modulated by levels of mitochondrial oxidative stress after experimental ozone exposure, we performed immunohistochemistry assays (2A; a. b, c, d, e, f). The median number of cells that showed cytoplasmic immunoreactivity to cytochrome c started to increase at 7 and 15 days. However, we observed that cytochrome c translocated to the nucleus at 30 days of exposure. In general, the median number of cells immunoreactive for cytochrome c increased significantly in the 15-, 30-, 60-, and 90-day exposure groups (*P* < 0.03) compared to the control group (Figure 2B). Western blot analysis was performed; however there was no statistically significant difference between the groups (data not shown).

### GFAP immunohistochemistry

**Figure 3:**
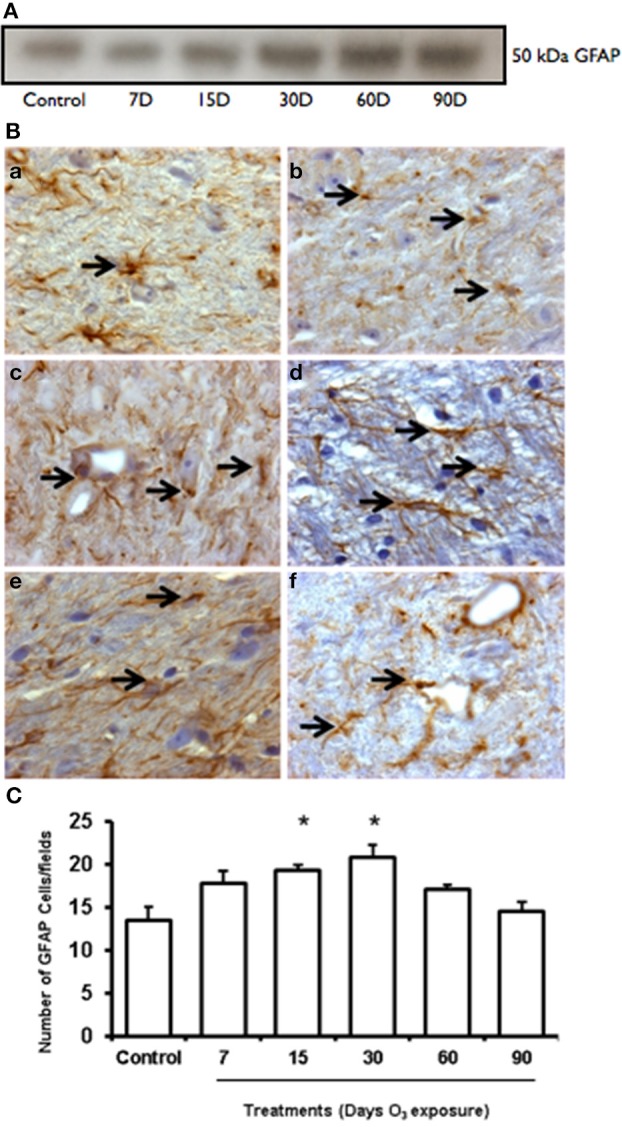
**Effct of ozone treatment on GFAP protein expression in the substantia nigra**. **(A)** Representative western blot of GFAP expression. **(B)** Light photomicrographs showing GFAP immunoreactivity (arrows) in the substantia nigra of the rats treated with air only (a), 7 days of ozone exposure (b), 15 days of ozone exposure (c), 30 days of ozone exposure (d), 60 days of ozone exposure (e), and 90 days of ozone exposure (f). Arrows show normal GFAP. 40X. In the ozone treatment groups, we observed changes in the size of astrocytes (c) and morphological changes. **(C)** The graph shows the effect of ozone treatment on the number of GFAP-positive cells in the substantia nigra. These changes were statistically significant for 15 and 30 days of treatment. The mean numbers (±SE) of GFAP-positive cells are depicted on the ordinate. The treatments are indicated under each bar (control group and 7, 15, 30, 60, and 90 days of ozone exposure); *n* = 6 per group. ^*^P < 0.05.

Western blot showed an increase of GFAP expression at 7, 15, 30, and 60 days of ozone exposure (Figure 3A). Normal astrocytes were observed in the GFAP immunohistochemistry in the control group. In the groups exposed to ozone for 7 (2b), 15 (2c), and 30 days (2d), we detected an increase in the number of immunoreactive cells, and for the 30- and 60-day (2e) exposure groups, the GFAP immunoreactivity levels in each cell increased. Particularly in the groups exposed to ozone for 30 and 60 days, GFAP increased in the processes of astrocytes. All astrocytes indicate astroglial reactivity. In the substantia nigra of animals exposed to 90 days of ozone, astrocytes showed morphological changes (Figure 3B). The number of astrocytes in the substantia nigra changed in the groups exposed to ozone for 15 (19 ± 0.6), 30 (20.75 ± 1.5), and 60 days (17 ± 0.6) compared to the control group (13 ± 1) (*P* < 0.01). The groups exposed to ozone for 15, 30, and 60 days had significantly more astrocytes than the group exposed to ozone for 90 days (*P* < 0.05) (Figure 3C).

### Microglial immunohistochemistry

**Figure 4:**
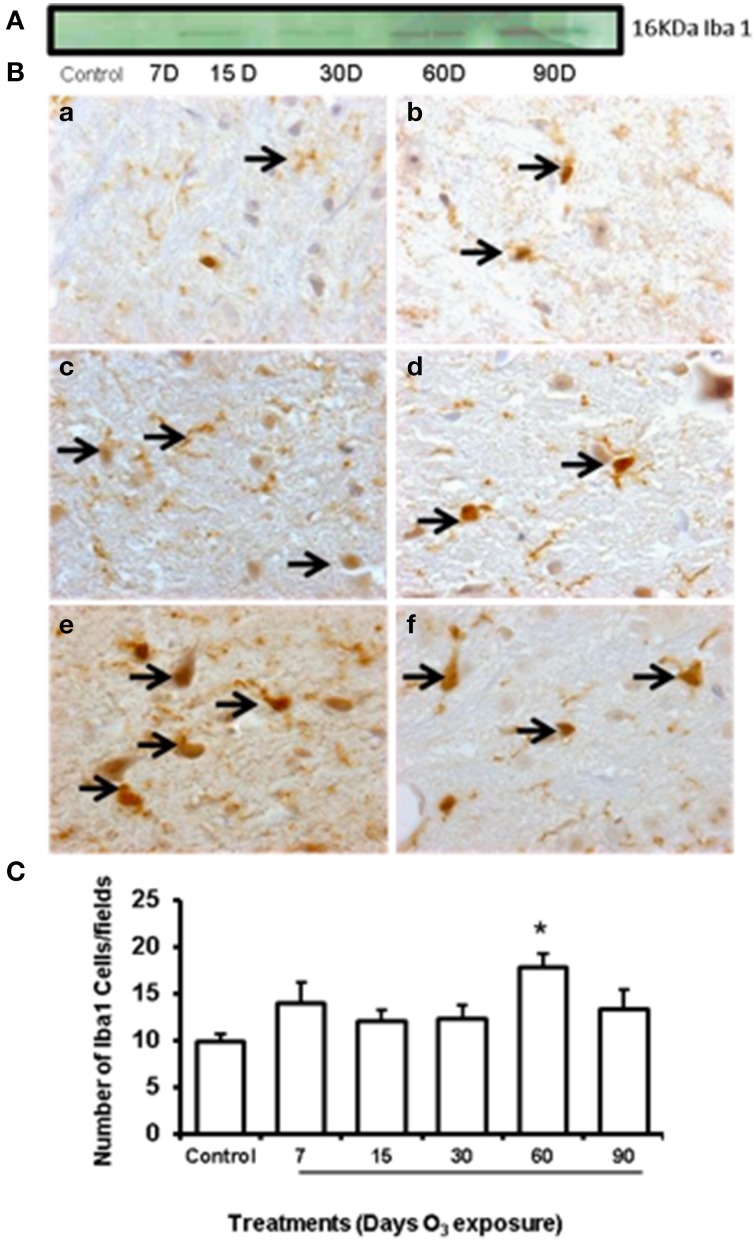
**Effect of ozone treatment on Iba-1 immunoreactivity in the substantia nigra**. **(A)** Representative western blot of the Iba-1 expression pattern. **(B)** Light photomicrographs show Iba-1 immunoreactivity in the substantia nigra of the rats treated with air only (a), 7 days of ozone exposure (b), 15 days of ozone exposure (c), 30 days of ozone exposure (d), 60 days of ozone exposure (e), and 90 days of ozone exposure (f). Arrows show microglia with normal Iba-1 immunoreactivity. 40x. We observed a phenotypical change in the microglia with ozone treatment. **(B)** The graph shows the effect of ozone treatment on the number of Iba-1-positive microglia in the substantia nigra **(C)**. The mean numbers (±SE) of Iba-1-positive microglia are depicted on the ordinate. The treatments are indicated under each bar (control group and 7, 15, 30, 60, and 90 days of ozone exposure); *n* = 6 per group. ^*^P < 0.05.

Western blot showed an increase of GFAP expression at 60 and 90 days of ozone exposure (Figure 4A). Microglial immunoreactivity for Iba-1 showed that the chronic oxidative stress induced by ozone produced phenotypical changes in microglia in the substantia nigra (Figure 4B). We measured a decrease of resting microglia beginning in the 7- and 15-day groups (b, c) and an increase of activated and phagocytic microglia in the 60-day group (e) (*P* < 0.05). However, the number of microglial cells increased at 60 days of ozone exposure and decreased at 90 days (Figure 4C).

### NFKB immunohistochemistry

**Figure 5:**
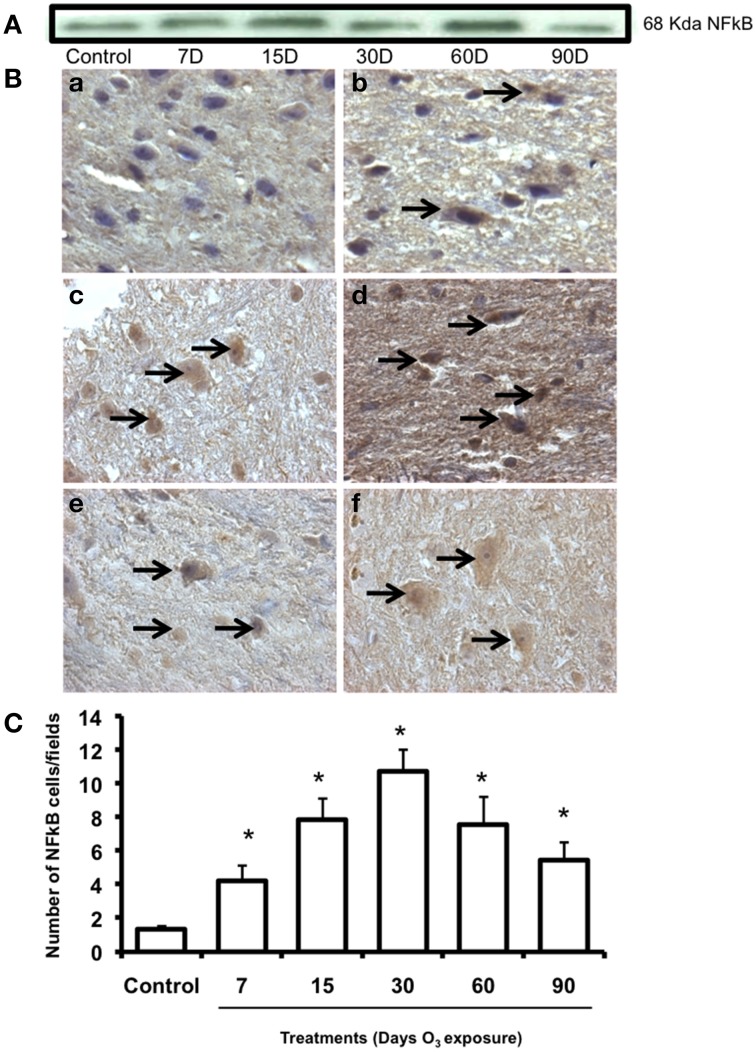
**Effect of ozone treatment on NFkB immunoreactivity in the substantia nigra**. **(A)** Representative western blot for NFkB expression. **(B)** Light photomicrographs show NFkB immunoreactivity in the substantia nigra of the rats treated with air only (a), 7 days of ozone exposure (b), 15 days of ozone exposure (c), 30 days of ozone exposure (d), 60 days of ozone exposure (e), and 90 days of ozone exposure (f). Arrows show cells with normal NFkB immunoreactivity. 40X. We observed an increase of immunoreactivity in the cell nucleus with ozone treatment and a decrease in the number of NFkB-positive cells in (e) and (f). **(C)** The graph shows the effect of ozone treatment on the number of NFkB-positive cells in the substantia nigra. The mean numbers (±SE) of NFkB-positive cells are depicted on the ordinate. The treatments are indicated under each bar (control group and 7, 15, 30, 60, and 90 days of ozone exposure); *n* = 6 per group. ^*^P < 0.05.

NFkB expression pattern was observed by western blot and showed an increase in protein expression in the 60-day exposure group and a decrease in the 90-day group with respect to the control group expression at 15 and 60 days of ozone exposure (Figure 5A). NFκB immunoreactivity (Figure 5B) showed an increase in nuclear translocation at 7 (b), 15 (c), and 30 days (d) of ozone exposure and decreases at 60 (e) and 90 (f) days The median number of cells in the substantia nigra with a nucleus immunoreactive for NFκB increased significantly (*P* < 0.003) in the groups exposed to ozone for 7, 15, and 30 days compared to the control group (Figure 5C). Compared to the 15-day group (*P* < 0.05), a significant decrease in the number of neurons was also found in the groups exposed to ozone for 60 and 90 days (Figure 5C). Data are presented as the mean of three independent experiments.

### COX-2 immunohistochemistry

**Figure 6:**
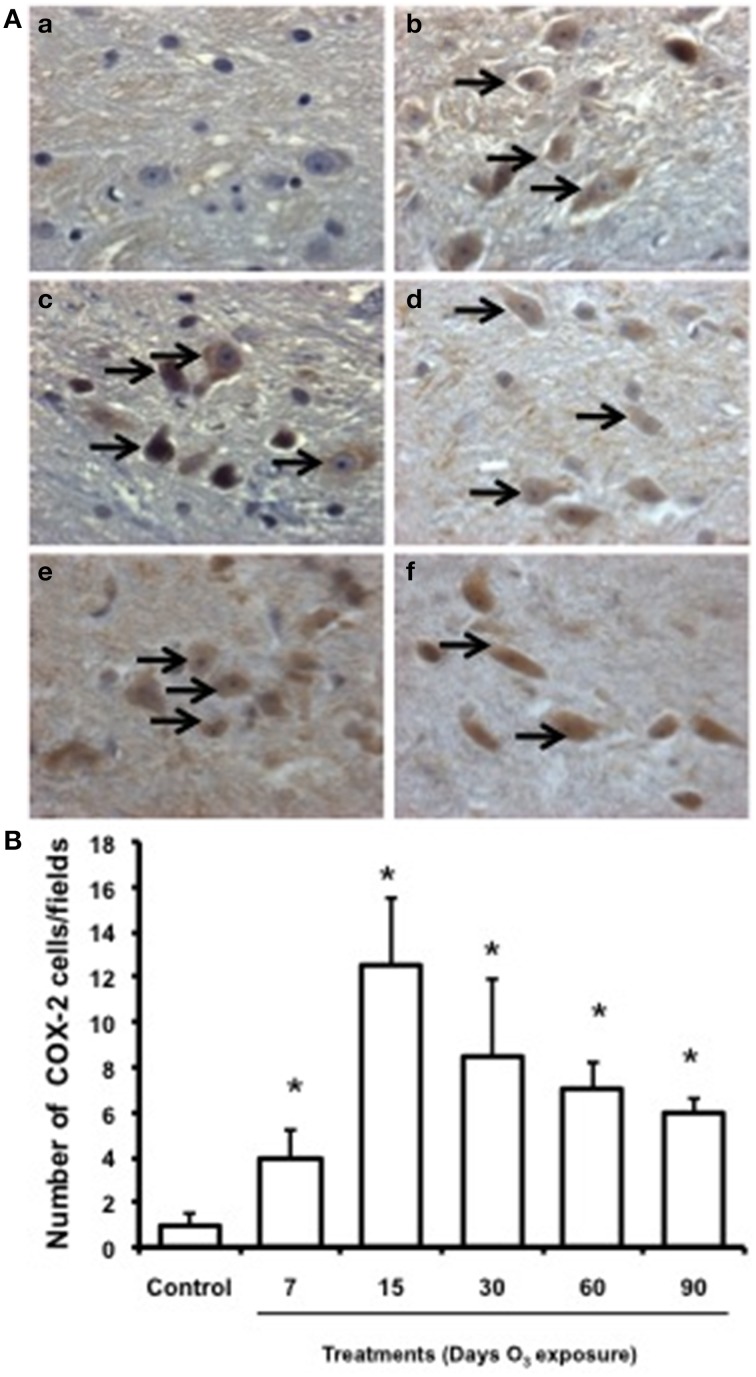
**Effect of ozone treatment on COX-2 expression in the substantia nigra**. **(A)** Light photomicrographs show COX-2 immunoreactivity in the substantia nigra of the rats treated with air only (a), 7 days of ozone exposure (b), 15 days of ozone exposure (c), 30 days of ozone exposure (d), 60 days of ozone exposure (e), and 90 days of ozone exposure (f). Arrows show cells with normal COX-2 immunoreactivity. 40x. We observed an increase of cellular immunoreactivity in the rats treated with ozone. **(B)** The graph shows the effect of ozone treatment on the number of COX-2-positive cells in the substantia nigra. Mean numbers (±SE) of COX-2-positive cells are depicted on the ordinate. The treatments are indicated under each bar (control group and 7, 15, 30, 60, and 90 days of ozone exposure); *n* = 6 per group. ^*^P < 0.05.

Low immunoreactivity for COX-2 was found in the substantia nigra of the control group. In the groups exposed to ozone for 7, 15, and 30 days, immunoreactivity for this enzyme increased. In the groups exposed for 60 and 90 days, marked immunoreactivity and a qualitative change in cell morphology were observed (Figure 6A). The number of substantia nigra cells per field that were immunoreactive for COX-2 (Figure 6B) was significantly different in the groups exposed to ozone for 7 (b), 15 (c), 30 (d), 60 (e), and 90 days (f) (*P* < 0.05) compared to the control group (a).

## Discussion

Previous results from our laboratory have shown that acute ozone exposure (0.8 ppm) causes oxidative stress, as shown by an increase in lipid peroxidation levels in different brain structures (Rivas-Arancibia et al., 2003, 2013; Angoa-Pérez et al., 2006). In addition to oxidative stress, acute ozone exposure causes neuronal morphological and ultrastructural changes (Rivas-Arancibia et al., 2013). Our previous results in this animal model have shown that groups chronically exposed to ozone for 7 and 15 days (0.25 ppm) recover from the damage 30 days after the ozone exposure. After 30 days of ozone exposure, the neurodegenerative process is irreversible and the brains of the rats show an increase of cellular and structural damage over time (4, 18).

In this study, rats were chronically exposed to 0.25 ppm because the transient effects of ozone are more closely related to accumulative daily exposure than to 1-h peak concentrations.

These results show the potential effect of ozone-based environmental pollution in the brain. Previous results have shown alterations in exploratory and freezing behaviors, which were detected in groups exposed to ozone compared to the control group (Dorado-Martínez et al., 2001; Rivas-Arancibia et al., 2003); this erratic behavior could be a consequence of the dopaminergic neuronal damage in the substantia nigra and striatum (Santiago-López et al., 2010). In this model, this damage is caused by a chronic state of oxidative stress, as indicated by the increase in protein oxidation levels in the groups exposed to ozone (Figure 1). Altered levels of oxidized proteins affect correct cell functioning. In this way, one of these altered proteins could be dopamine. Dopamine has an oxidative metabolism that makes dopaminergic neurons and their fibers especially sensitive to oxidative stress. A chronic state of oxidative stress induced by ozone exposure causes dopamine oxidation, produces dopamine quinones in the striatum, and increases the oxidative process as we have previously shown (Rivas-Arancibia et al., 2003; Santiago-López et al., 2010). Dopamine and dopamine quinones may react with cysteine, producing free radicals and dehydrobenzothiazines and depleting antioxidant defenses (Rivas-Arancibia et al., 2004). This process can contribute to progressive cell death, as cytochrome c release from mitochondria occurs in this model (Figure 2) (Rodríguez-Martínez et al., 2013).

In this study, we show that for 15–90 days of ozone treatment, there was a translocation of cytochrome c from mitochondria to the cytosol. The cytochrome c translocation could be correlated with the mitochondrial protein oxidation previously reported by Rodríguez-Martínez et al. (2013), suggesting that the release of cytochrome c after ozone treatment is mediated in part by mitochondrial oxidative stress-dependent mechanisms (Fujimura et al., 1998, 1999; Ramsden et al., 2001).

Previous data has supported the role of cytochrome c release as the initiator of the mitochondrial apoptotic pathway (Fujimura et al., 2000; Sugawara et al., 2000). The release of cytochrome c results in additional ROS production through inhibition of the respiratory chain (Li et al., 1997). These events promote a vicious cycle of increased cytochrome c release followed by increased mitochondrial ROS production, which may be maintained.

Finally, Liu et al. (1997) and Lewén (2001) have demonstrated that severe injury with marked ROS stress leads to necrotic cell death, whereas milder ROS stress activates apoptotic pathways. Our results suggest that cytochrome c release could lead to overall cell death or apoptosis. However, which mechanism is activated in our model remains to be elucidated.

In another way, GFAP has been widely used is as a molecular marker for gliosis. GFAP is an intermediate filament protein that mediates the acquisition of the fibrotic phenotype of reactive astrocytes. There are several studies that have shown an increase in its expression during aging and its overexpression in oxidative stress states (Morgan, 1997; Cai et al., 1998; Lewén, 2001). Our results show that oxidative stress produces morphological alterations and cell death, causing an increase of GFAP expression from 15 until 90 days of treatment. A significant increase in the number of GFAP cells in the substantia nigra was also found after 15 and 30 days of treatment, but this response decreased in the 60- and 90-day groups. There are many reports that have demonstrated phenotypic changes in astrocytes accompanied by the increase of proinflammatory cytokines and a loss of the microglial neuroprotective function in different neurodegenerative diseases (Tacconi et al., 1991; Choi and Yu, 1995; Zhang et al., 2005; Garden and Möller, 2006; Gerhard et al., 2006; Solito and Sastre, 2012). However, more studies are needed to demonstrate the microglia loss function in this oxidative stress model. Previously, we have reported that there are irreversible changes and neurodegenerative processes that start after 30 days of ozone exposure. Therefore, we could suggest that this cellular response has been inhibited by the oxidative stress state induced by ozone. However, further studies are needed to probe this hypothesis.

The TH immunohistochemistry previously reported by our group showed qualitative changes in neuronal shape and size, the loss of dendrites, the presence of cytoplasmic vacuoles, damage to the neuropil and significant decreases in the number of neurons in the substantia nigra (Pereyra-Muñoz et al., 2006). The neurodegenerative process has been linked to inflammatory processes, as we have seen in astrocytic changes (Figures 3, 4). Together with the increase in Iba-1 protein at 60 and 90 days of exposure (Figure 4), the phenotypical changes in the microglia that transitioned from resting microglia to activated and phagocytic microglia suggests an alteration of the activation of immune responses induced by ozone exposure. There are different immune responses within different cell types, especially with respect to the microglia and macrophages that may contribute to inflammation and subsequent cell death. Moreover, these changes are correlated with alterations in the number of NFkB immunoreactive cells and NFkB protein expression level, which was observed following 7–90 days of exposure (Figure 5).

Exposure to ozone causes oxidative stress, which triggers the inflammatory response and there are many pathways by which inflammation is activated by reactive species (Choi and Yu, 1995; Vijitruth et al., 2006; Orre et al., 2014). One of these pathways is the activation of membrane kinases that activate NFkB, which causes an increase in the production of inflammatory cytokines.

NFkB is a transcription factor thought to be regulated by oxidative stress and has been recognized for its role in the induction of inflammatory responses (Schreibelt Kooij et al., 2007). However, this activation of NFkB can result in enhanced de novo synthesis of proteins that both confer protection and cause death (Schreibelt Kooij et al., 2007; Orre et al., 2014). In this study, we observed a large number of cells immunoreactive for NFkB as a result of treatment; however, the number decreased after 60 and 90 days of treatment. Based on these results, we can suggest that compared to the control, a large number of cells respond to ozone treatment. We can also suggest that altered immune responses are activated by oxidative stress. These immune responses could trigger pro-inflammatory processes activated by NFkB through the non-canonical pathway (Helenius, 1996; Morgan and Liu, 2010). The increase in COX-2 immunoreactivity supports this hypothesis (Figure 6).

Oxidative stress caused by low-dose exposure to ozone activates COX-2, which is involved in the production of pro-inflammatory prostaglandins (Martínez-Canabal et al., 2008). This response can be activated by the non-canonical NFkB pathway (Orre et al., 2014). These results suggest that the oxidative stress itself is activating inflammatory responses when there are alterations produced by chronic exposure to ozone, and this type of response could be involved in cell death mechanisms. However, more mechanistic studies are needed to demonstrate the importance of these alterations and what type of responses are activated by NFkB.

The physiological inflammatory response is an auto-limited response and is evoked to repair an altered microenvironment. This regulated response was initiated after 7 and 15 days of ozone exposure in this experiment, but when the neurodegenerative process is irreversible (30, 60, and 90 days of ozone exposure), dysregulation of the inflammatory response occurs, which alters the blood-brain barrier (Rivas-Arancibia et al., 2013), triggering all inflammatory processes. It has been suggested that the inflammatory response could cause alterations in the blood-brain barrier in the zone of injury (Morgan and Liu, 2010). During this process, there is a loss of endothelial cell function and capillary changes in the structures affected (Rivas-Arancibia et al., 2013). In addition to the alterations of the tight junctions of endothelial cells, pericytes and the ends of the astrocyte terminals are altered as well (Schreibelt Kooij et al., 2007). These alterations cause the barrier to be more permeable and less selective, allowing substances that require transporters to cross the barrier more easily (Rivas-Arancibia et al., 2013). When a brain-damaging process is accompanied by inflammation, substances and cells are found that are not normally present in these sites. This hypothesis has been used to explain the progressive damage in Parkinson's disease (Morgan and Liu, 2010; Przedborski, 2010).

The presence of dopamine quinones in the substantia nigra and striatum in humans has been reported (Gerhard et al., 2006). Dopamine oxidation, inflammation, oxidative stress, and dysfunction of the ubiquitin proteasome system are factors that, as a whole, contribute to the pathogenesis and progression of Parkinson's disease (Helenius, 1996; Asanuma et al., 2004; Martínez-Canabal et al., 2008; Miyazaki and Asanuma, 2008).

In conclusion, our model shows that ozone exposure induces oxidative stress. The state of chronic oxidative stress could itself generate progressive cell death in the substantia nigra depending on the duration of ozone exposure. This process occurred together with a dysregulation of the inflammatory response, dopamine oxidation, and cell death, producing a vicious cycle of the generation of an extensive oxidative stress state and the incapacity of antioxidant systems to counteract it, as could be happening in Parkinson's disease.

### Conflict of interest statement

The authors declare that the research was conducted in the absence of any commercial or financial relationships that could be construed as a potential conflict of interest.
